# SLC22A3 rs2048327 Polymorphism Is Associated with Diabetic Retinopathy in Caucasians with Type 2 Diabetes Mellitus

**DOI:** 10.3390/biomedicines11082303

**Published:** 2023-08-18

**Authors:** Emin Grbić, Mojca Globočnik Petrovič, Ines Cilenšek, Danijel Petrovič

**Affiliations:** 1 Department of Physiology, Faculty of Medicine, University of Tuzla, 75000 Tuzla, Bosnia and Herzegovina; emin.grbic@untz.ba; 2 Clinic of Ophthalmology, Ljubljana University Medical Centre, 1000 Ljubljana, Slovenia; mojca.globocnik@kclj.si; 3 Institute of Histology and Embryology, Faculty of Medicine, University of Ljubljana, 1000 Ljubljana, Slovenia; ines.cilensek@mf.uni-lj.si

**Keywords:** SLC22A3, rs2048327, diabetic retinopathy, immunohistochemistry

## Abstract

The Solute Carrier Family 22 Member 3 (SLC22A3) is a high-capacity, low-affinity transporter for the neurotransmitters norepinephrine, epinephrine, dopamine, serotonin, and histamine. SLC22A3 plays important roles in interorgan and interorganism small-molecule communication, and also regulates local and overall homeostasis in the body. Our aim was to investigate the association between the rs2048327 gene polymorphism and diabetic retinopathy (DR) in Slovenian patients with type 2 diabetes mellitus (T2DM). We also investigated SLC22A3 expression in the fibrovascular membranes (FVMs) of patients with proliferative DR (PDR). Our study involved 1555 unrelated Caucasians with T2DM with a defined ophthalmologic status: 577 of them with DR as the study group, and 978 without DR as the control group. The investigated polymorphisms were genotyped using the KASPar genotyping assay. The expression of SLC22A3 (organic cation transporter 3—OCT3) was examined via immunohistochemistry in human FVM from 16 patients with PDR. The C allele and CC genotype frequencies of the rs2048327 polymorphism were significantly higher in the study group compared to the controls. The logistic regression analysis showed that the carriers of the CC genotype in the recessive genetic models of this polymorphism have a 1.531-fold increase (95% CI 1.083–2.161) in the risk of developing DR. Patients with the C allele of rs2048327 compared to the homozygotes for the wild type T allele exhibited a higher density of SLC22A3 (OCT3)-positive cells (10.5 ± 4.5/mm^2^ vs. 6.1 ± 2.7/mm^2^, respectively; *p* < 0.001). We showed the association of the rs2048327 *SLC22A3* gene polymorphism with DR in a Slovenian cohort with type 2 diabetes mellitus, indicating its possible role as a genetic risk factor for the development of this diabetic complication.

## 1. Introduction

Diabetic retinopathy (DR) is one of the leading complications of diabetes that can cause visual impairment or blindness. A third of people with diabetes worldwide have DR, and a third of those people have retinopathy that could result in vision loss [1]. The development and progression of diabetic retinopathy are influenced by the type and duration of diabetes. Type 1 diabetes carries a higher relative risk of developing diabetic retinopathy than type 2, while type 2 diabetes carries a higher prevalence of diabetic macular edema [2]. Changes in the rheological properties of blood, as well as changes in the wall of retinal blood vessels, lead to capillary occlusion, hyperpermeability, and retinal ischemia. The combinations of these changes with inflammatory changes lead to the appearance and development of DR, with characteristic histological changes, the loss of pericytes, and the thickening of the basilar membrane. Microaneurysms, or locations where the capillary wall balloons outward, are pathognomonic [3], alongside retinal hemorrhages, macular edema, and retinal neovascularization.

In recent years, the role of diabetic retinal neurodegeneration is increasingly evident, not only as a biomarker for DR but also as a causal factor in the development of DR [4,5,6]. Retinal ganglion cell apoptosis, as the most important feature of neurodegeneration, occurs early in the disease course, presented as a thinner retinal inner layer by OCT imaging [7,8,9,10,11].

There are numerous biochemical signaling pathways at play among which the role of oxidative stress is crucial. Kang and Yang showed that hyperglycemia can cause oxidative stress through a series of pathways, including the polyol, protein kinase C, and hexosamine pathways, as well as an increased expression of advanced glycation end products (AGEs) and their receptors. This imbalance in cellular redox homeostasis is further exacerbated by the abnormal activity of nuclear factors like highly activated NF-κB, and the diminished activity of Nrf2, as well as hyperglycemia-induced mitochondrial dysfunction. These factors contribute to the overproduction of reactive oxygen species (ROS) in DR [12]. An overproduction of ROS leading to oxidative stress can upset the balance of the metabolic system and cause the dysfunction of the retinal neurovascular unit, resulting in apoptosis, inflammation, and the degeneration of both the vasculature and neurons. It may also promote the growth of new blood vessels (neovascularization). Additionally, oxidative stress can lead to epigenetic changes and alter the expression of genes involved in signal transduction [13,14,15].

Organic cation transporter 3 (OCT3, SLC22A3) is a high-capacity, low-affinity transporter for the neurotransmitters norepinephrine, epinephrine, dopamine, serotonin, and histamine. Corticosterone has a direct impact on inhibiting the OCT3-mediated transport of these neurotransmitters. Corticosteroid hormones can affect gene expression by acting on intracellular glucocorticoid receptors (GR) and mineralocorticoid receptors (MR), leading to a wide range of physiological and behavioral responses. In addition, corticosteroid hormones also have rapid effects on physiology and behavior through non-genomic mechanisms. Some of these mechanisms are dependent on GR or MR, while others are independent of these receptors. One such GR-independent mechanism involves the inhibition of monoamine transport mediated by “uptake2” transporters, including OCT3, as a result of corticosteroid action [16].

Dopamine and histamine are important neurotransmitters and neuromodulators in the retinal neurons [17,18].

Dopamine supplementation in diabetic rodent models prevents visual loss because dopamine levels in the retina are decreased [19,20]. According to a preclinical study in diabetic patients, neurodegeneration can be detected early on by electroretinography and treated with L-dopa to prevent it from progressing to clinically recognized retinopathy [21]. Dopamine was found to exert protective effects against microvascular leaking and pericyte and endothelial loss, as well as to inhibit hyperglycemia-induced oxidative stress and mitochondrial dysfunction in HRECs and mouse retinas; therefore, it affects the development of diabetic vasculopathy as well [22].

The Solute Carrier Family 22 Member 3 (SLC22A3 also known as organic cation transporter 3—OCT3, gene ID: 6581, OMIM: 604842, HGNC: 10967) is a protein-encoding gene located on chromosome 6 on the q arm at position 25.3 [23]. The protein encoded by this gene belongs to the SLC22 transporter family. The neuroendocrine, growth factor–cytokine, and other homeostatic systems, along with SLC22 transporters, play important roles in interorgan and interorganism small-molecule communication. These systems also regulate local and overall homeostasis in the body [24]. The SLC22 transporter family is divided into two major clades: OAT—organic anion transporters; OCT—organic cation transporters. Each of these clades can be further divided into three subclades, designated as OAT, OAT-like, OAT-related, OCT, OCTN (organic cation/carnitine transporter), and OCT/OCTN-related. The OCT subclade has three known members: OCT1, OCT2, and OCT3 (SLC22A1, SLC22A2, and SLC22A3). OCT3 is more widely expressed than OCT1 and OCT2. OCT3 expression is widespread and can be found in the skeletal muscle, heart, brain, placenta, cornea, retina, iris, liver, kidney, and vascular endothelial cells [24,25,26,27].

To date, the exact mechanism of action of OCT3 has not been fully elucidated. However, OCT3 is associated with liver fibrosis, hepatocellular carcinoma, colorectal carcinoma, and altered drug pharmacokinetics [28,29,30,31].

A few studies found an association between the rs2048327 polymorphism of the *SLC22A3 (OCT3)* gene and CHD (coronary heart disease) in the Iranian population, and CVD (cardiovascular disease) and low HDL concentration in the Canadian population [32]. In contrast to these studies, studies on the Chinese Han population found no association between the rs2048327 polymorphism and coronary heart disease. The same findings were supported by a second study that also included this population [33]. Possible reasons for the opposite results of the aforementioned studies may be due to the effects of epigenetic and environmental factors in the aforementioned populations. However, data on the polymorphism rs2048327 and its association with the DR, and prognostic and therapeutic significance are lacking.

The objective of this study was to investigate the association between the polymorphisms of the *SLC22A3* gene and the development of DR among patients with type 2 diabetes in the Slovenian population (Caucasians). Moreover, we investigated the association of the rs2048327 polymorphism of SLC22A3 gene expression in the FVMs of patients with T2DM and PDR.

## 2. Materials and Methods

### 2.1. Patients

In our case–control study, were enrolled 1555 unrelated Caucasians with T2DM, with a defined ophthalmologic status. The current American Diabetes Association criteria were used to identify patients with T2DM [34]. Between January 2010 and January 2023, participants were collected from the University Medical Center Ljubljana Diabetic Outpatient Clinic and Eye Clinic. After pupil dilation, as aforementioned, a senior eye specialist (M.G.P.) performed a fundus examination [35]. The study group consisted of 1555 subjects: 577 subjects with DR (cases) and the control group of 978 subjects with T2DM of more than a 10-year duration who had no clinical signs of DR.

Patients with overt nephropathy were not included in the study to avoid the confounding effect of impaired kidney function. Moreover, patients with other ocular diseases were excluded. The study was carried out in accordance with the Helsinki Declaration and received approval from the National Ethical Committee (number 118/12/2011). Following the acquisition of informed consent for study participation, a thorough interview was conducted.

### 2.2. Biochemical Analyses

Standard biochemical techniques were used to measure glucose, creatinine, total cholesterol, low-density lipoproteins (LDL), high-density lipoproteins (HDL), and triglycerides.

### 2.3. Genotyping

Using a Qiagen isolation kit, genomic DNA was extracted from 100 μL of whole blood. The rs2048327 polymorphism of the SLC22A3 gene was genotyped by LGC Biosearch Technologies using their own novel, fluorescence-based, competitive, allele-specific PCR (KASPar) assay. The details of the method used can be found at http://running-KASP-on-ABI-StepOne-and-StepOnePlus.pdf (accessed on 15 July 2023).

### 2.4. Immunohistochemistry

In this study, we investigated the expression of SLC22A3 in fibrovascular membranes (FVMs) obtained from 25 patients (mean age: 62.5 ± 14.3 years; 13 men and 12 women) with type 2 diabetes mellitus (T2DM) and proliferative diabetic retinopathy (PDR) during pars plana vitrectomy. A pars plana vitrectomy with fibrovascular membrane peeling was performed by an experienced surgeon (M.G.P.). Formalin-fixed paraffin-embedded (FFPE) tissue sections of the FVMs were prepared by cutting consecutive 5 μm sections from each paraffin block and mounting them on glass slides. Deparaffinization and dehydration were carried out using a series of alcohol solutions. To detect the SLC22A3 protein in our tissue samples, we performed immunohistochemistry using the i-View method with the Invitrogen SLC22A3 antibody (MA5-36158) on the Ventana Roche diagnostic system (Tucson, AZ, USA). To ensure the reliability of the staining procedure, positive and negative controls were included. Kidney tissue sections known to express SLC22A3 were used as positive controls, while brain tissue sections, which exhibited minimal or absent SLC22A3 expression, were used as negative controls.

Microscopic analysis was conducted to evaluate SLC22A3 expression in the fibrovascular membranes. The presence or absence of specific staining in the fibrovascular membranes was compared to the positive and negative control tissues. A quantitative assessment of SLC22A3 expression was performed by manually identifying and marking SLC22A3-positive cells. The area containing SLC22A3-positive cells was delineated, and the numerical areal density of SLC22A3-positive cells was calculated as the number of positive cells per square millimeter (mm^2^) of the marked area. Data obtained from the stained tissue sections were analyzed qualitatively, and the expression and distribution of SLC22A3 in fibrovascular membranes were assessed. A statistical analysis was conducted using appropriate methods to determine the significance of any observed differences.

### 2.5. Statistical Analysis

The SPSS program for Windows version 24 (SPSS Inc., Chicago, IL, USA) was used for the statistical analyses. An unpaired Student’s *t*-test was used to compare continuous clinical data, and the chi-square test was used to compare discrete variables. For continuous variables, data were presented as mean SD, while, for categorical variables, the number and percentage of patients were used. In the study, the normality of data distribution was ascertained using the Shapiro–Wilk test. Continuous variables were compared using an unpaired Student’s *t*-test when normally distributed, and the Mann–Whitney U test when asymmetrically distributed. Continuous variables were reported as mean ± standard deviation when normally distributed, and as median values (interquartile range (IQR)) when asymmetrically distributed.

Additionally, a multiple logistic regression was used to incorporate all variables that by univariate analysis demonstrated significant differences. Statistical significance was set at *p* < 0.05.

## 3. Results

The clinical characteristics of the two groups are summarized in Table 1. The following parameters showed a statistically significant difference between the groups: waist circumference (*p* < 0.006), duration of T2D (*p* < 0.001), BMI (*p* < 0.041), insulin therapy (*p* < 0.001), CVD (*p* < 0.001), diabetic neuropathy (*p* < 0.001), diabetic foot (*p* < 0.001), and S-HbA1c (*p* < 0.001). However, there were no significant differences between the groups with respect to age, sex, systolic blood pressure, diastolic blood pressure, active smokers, fasting glucose, total cholesterol, HDL-cholesterol, LDL-cholesterol, and triglycerides. Patients in the case group had a larger waist circumference, a longer duration of T2D, a higher use of insulin, a higher percentage of diabetic complications such as diabetic neuropathy and diabetic foot, and higher HbA1c values compared to the control group.

Data on the genotype distribution and allele frequency of the rs2048327 polymorphism of the *SLC22A3* gene are shown in Table 2. The univariate analysis showed a statistically significant difference in genotype distribution (*p* < 0.008), as well as in allele frequency (*p* < 0.003) between the case group and controls. The *SCL22A3* genotype distribution in cases and controls did not deviate significantly from Hardy–Weinberg equilibrium.

We used logistic regression analysis to assess whether the rs2048327 polymorphism was independently associated with DR after adjusting for waist circumference, duration of T2D, BMI, diabetic neuropathy, and HbA1c. The results in the two genetic models, co-dominant (*p* < 0.013) and recessive (*p* < 0.016), indicate the existence of a statistically significant association (Table 3). The statistical strength of the study was 0.80.

**Figure 1:**
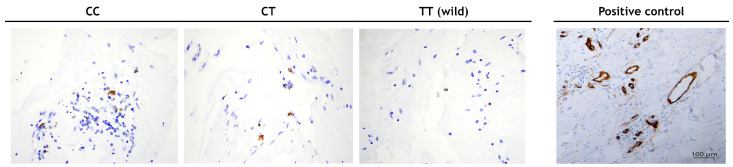
SLC22A3-positive cells in fibrovascular membranes from patients with PDR.

In the FVMs of diabetic patients with PDR, a significantly higher numerical areal density of OCT-3-positive cells (Figure 1) was found in patients with the C allele of rs2048327 (CC + TC genotypes) compared to the homozygotes for the wild type T allele (10.5 ± 4.5/mm^2^ vs. 6.1 ± 2.7/mm^2^, respectively; *p* < 0.001).

## 4. Discussion

The purpose of our study was to investigate the association between the rs2048327 of the *SLC22A3* gene and DR in the Slovene Caucasians with T2DM. Logistic regression analysis showed that there is an increased risk in carriers of the rs2048327 polymorphism for DR in two genetic models (co-dominant and recessive). The findings of our study should be compared to those of similar or related studies, and the potential for their use in clinical practice should be investigated.

This is the first report of any *SLC22A3* polymorphism on DR, a diabetic microvascular complication in T2DM. Many epidemiological studies have been conducted worldwide in the last few decades regarding the relationship between the rs2048327 *SLC22A3* gene polymorphism and other microvascular and vascular complications, such as diabetic nephropathy, cardiovascular disease (CVD), an coronary heart disease (CHD) [36].

In a study of unrelated Finnish patients with T1DM, an association was found between *SLC22A3* rs2048327 and diabetic nephropathy (DN) in men (*p* < 0.03) [36]. In their study, patients were divided into two samples: sample 1 (1086 patients) for the primary association analysis, and sample 2 (1252 patients) for replication. Patients were selected from all over Finland as part of the FinnDiane study. They also genotyped 165 non-diabetic Finns across the country. In the study, the age of onset of type 1 diabetes, the time of initiation of insulin therapy, and the fasting C peptide level were measured. According to the renal status, the patients were further divided into three groups, patients with normo-, micro-, and macroalbuminuria. In comparison with our study, the patients were younger and had a longer duration of diabetes, which is expected considering that type 1 diabetes mellitus appears earlier compared to in our subjects who have type 2 diabetes. It is also noticeable that out of the patients from the abovementioned study, 79.3% had hypertension in the microalbuminuria subgroup, and as many as 97.5% did in the macroalbuminuria subgroup. Similar to our study, in the aforementioned study, the group of cases includes mostly men. Regarding the rs2048327 *SLC22A3* gene polymorphism, by stratifying patients into those with HbA1c < 8.4% and HBA1c > 8.4%, an association with DN in men with HbA1c ≥ 8.4% was found, as well as allele frequency (*p* < 0.03), and genotype distribution (*p* < 0.04).

In a study on the Iranian population, which included 453 CHD patients and 453 non-CHD controls, the *SLC22A3* rs2048327 was significantly associated with an increased risk of CHD. A difference was found during the stratification analysis by gender, in male subjects from the case group compared to the control group, and in two genetic models: the additive (OR = 2.45; 95% CI: 1.17–5.11; *p* = 0.017) and recessive model (OR = 2.47; 95% CI: 1.21–5.06; *p* = 0.013). They also found that HDL concentration was 3.5 times lower in rs2048327 carriers [37]. In relation to our research, there is a difference in MAF frequency (minor allele frequency) between Iran and Slovenia (C allele frequency 29% and 35%). Also, in their research, no analysis of glycemic status was performed; but, despite this, the effect of rs2048327 on blood vessels was proven in the carriers of the C allele. Assuming that the effect of the rs2048327 polymorphism both in them and in us shows a possible association with changes in the blood vessels, it is possible that in our study there were changes in the blood vessels of the retina in the carriers of the C allele, with the influence of inadequately controlled glycemic status, which could ultimately favor the emergence and development of DR. Similar to the previous study, in a study on the Pakistani population, with 663 subjects of whom 78% were male, SLC22A3/rs2048327 showed an association with coronary stenosis (*p* < 0.045) in an additive genetic model [38]. In the aforementioned study, as in the previous two, there is a noticeably higher percentage of male respondents. The influence of gender is not negligible in the development of the mentioned events in conjunction with the rs2048327 polymorphism. Unfortunately, in the aforementioned studies, we do not have data on the diabetic status of the subjects. It is suggestive that in the mentioned studies, the rs2048327 polymorphism has an impact on changes in the blood vessels, and therefore we believe that it is possible that the mentioned polymorphism can also cause changes in retinal blood vessels, which could ultimately lead to DR in people with T2DM.

Contrary to previous studies, in the research on the Chinese Han population, no association of the rs2048327 polymorphism with the occurrence of CAD was found in any genetic model (recessive *p* = 0.09; dominant *p* = 0.84; additive *p* = 0.44) [39]. A similar finding was found in a study of Hispanics on the influence of the rs2048327 polymorphism on the occurrence of myocardial infarction (*p* = 0.93) [40]. Also, in another study on the Chinese Han population, a lower risk of developing CAD was found in carriers of the rs2048327 polymorphism (*p* = 0.016) [33]. The variance in sample size and population structure, failure to account for confounding factors, racial and ethnic differences, and the diversity of genetic and environmental backgrounds are some of the possible explanations for inconsistent results between populations. Additionally, the effects of population genotypes may vary among populations.

Additionally, our results suggest that the expression of SLC22A3 (OCT-3) is affected by the presence of the OCT-3 polymorphism (rs2048327), since the numerical areal density of rs2048327-positive cells was significantly higher in FVMs from DR patients carrying the C allele of rs2048327 compared to DR patients with the T allele, emphasizing the role of OCT-3 in the development of DR. To our knowledge, this is the first paper that investigated the expression of the OCT3 protein in the human retina. A similar paper was published analyzing the mouse retina [41]. A possible reason for the lack of works on this topic in humans is the difficulty in collecting samples for immunohistochemical analysis.

Our cross-sectional case–control study does, however, have some limitations. First of all, a relatively small but ethnically homogeneous cohort of cases and controls from Slovenia served as the research population. Secondly, in our study, there were differences in several parameters (for example, waist circumference, duration of T2D, BMI, diabetic neuropathy, HbA1c). By including each of these parameters in the logistic regression model, we attempted to reduce the impact of these differences. Thirdly, only one polymorphism of the SLC22A3 gene was studied. Therefore, more detailed research on larger samples with ethnically diverse subjects are needed to elucidate its role in the DR.

## 5. Conclusions

In our cross-sectional case–control study, we showed the association of the rs2048327 *SLC22A3* gene polymorphism with diabetic retinopathy in a Slovenian cohort with type 2 diabetes mellitus. In order to confirm the influence of the rs2048327 polymorphism on the occurrence and development of diabetic retinopathy, studies with a larger sample are necessary, as well as immunohistochemical analyses of the presence of the rs2048327 polymorphism on the retina in different populations.

## Figures and Tables

**Table 1:** Clinical and laboratory characteristics of patients with DR (cases) and without DR (controls).

	Case (N = 577)	Control (N = 978)	*p* Value
Sex [M]	300 (52.0%)	541 (55.3%)	0.2
Age [years]	65.11 ± 8.91	64.77 ± 9.66	0.53
Waist circumference [cm]	108.39 ± 13.03	105.17 ± 12.32	0.006
Duration of T2D [years]	18.00 (13.00–25.00)	12.00 (8.00–16.00)	<0.001
Duration of DR [years]	11 (10.00–25.00)		
SBP [mmHg]	148.68 ± 21.26	148.24 ± 20.17	0.69
DBP [mmHg]	84.05 ± 10.22	83.49 ± 10.69	0.32
BMI	29.49 ± 4.88	29.99 ±4.47	0.041
Active smokers	54 (9.4%)	119 (12.2%)	0.089
Insulin therapy	432 (74.9%)	387 (39.6%)	<0.001
CVD	118 (20.5%)	352 (36.0%)	<0.001
Diabetic neuropathy	112 (19.4%)	96 (9.8%)	<0.001
Diabetic foot	41 (11.9%)	0 (0.0%)	<0.001
S-HbA1c [%]	7.90 (7.00–8.83)	7.50 (6.80–8.30)	<0.001
S-fasting glucose [mmol/L]	8.50 (6.70–10.50)	8.20 (6.80–9.80)	0.065
S-total cholesterol [mmol/L]	4.80 (4.00–5.60)	4.70 (4.00–5.70)	0.65
S-HDL [mmol/L]	1.10 (1.00–1.35)	1.10 (1.00–1.40)	0.27
S-LDL [mmol/L]	2.70 (2.10–3.50)	2.70 (2.10–3.40)	0.53
S-TG [mmol/L]	1.80 (1.20–2.50)	1.70 (1.20–2.60)	0.72

Abbreviations: SBP—systolic blood pressure; DBP—diastolic blood pressure; BMI—body mass index; CVD—cardiovascular diseases; HbA1c—glycated hemoglobin; S-HDL—serum-high-density lipoprotein; S-LDL—serum-low-density lipoprotein; S-TG—serum–triglycerides.

**Table 2:** Genotype and allele frequencies of the rs2048327.

*SLC22A3*_rs2048327	Case (N = 577)	Control (N = 978)	*p* Value
Genotypes			
CC	85 (14.7%)	100 (10.2%)	0.008
CT	284 (49.2%)	466 (47.6%)	
TT	208 (36.0%)	412 (42.1%)	
Alleles			
C (MAF)	454 (39.3%)	666 (34.0%)	0.003
T	700 (60.7%)	1290 (66.0%)	
HWE (*p* value)	0.45	0.06	
dominant			
CC + CT	369 (64.0%)	566 (57.9%)	0.018
TT	208 (36.0%)	412 (42.1%)	
recessive			
CC	85 (14.7%)	100 (10.2%)	0.008
CT + TT	492 (85.3%)	878 (89.8%)	

Abbreviations: HWE—Hardy–Weinberg equilibrium; MAF—minor allele frequency.

**Table 3:** Association between rs2048327 and DR.

***SLC22A3*** **_rs2048327**	**CASES/CTRLs**	**Adjusted OR (95% CI)**	***p*** **Value**
co-dominant			
CC vs. TT	85/100 vs. 208/412	1.609 (1.104–2.345)	0.013
CT vs. TT	284/466 vs. 208/412	1.099 (0.860–1.403)	0.45
dominant			
[CC + CT] vs. TT	369/566 vs. 208/412	1.185 (0.938–1.499)	0.15
recessive			
CC vs. [CT + TT]	85/100 vs. 492/878	1.531 (1.083–2.161)	0.016

Adjusted for waist circumference, duration of T2D, BMI, diabetic neuropathy, S-HbA1c [%].

## Data Availability

The data presented in this study are available on request from the corresponding author. The data are not publicly available due to privacy restrictions.
